# Monitoring Lower Back Activity in Daily Life Using Small Unintrusive Sensors and Wearable Electronics in the Context of Rheumatic and Musculoskeletal Diseases

**DOI:** 10.3390/s21196362

**Published:** 2021-09-23

**Authors:** Mathieu Baijot, Robert Puers, Michael Kraft

**Affiliations:** 1 MNS, Department of Electrical Engineering (ESAT), University of Leuven, 3001 Leuven, Belgium; mathieu.baijot@kuleuven.be; 2 MICAS, Department of Electrical Engineering (ESAT), University of Leuven, 3001 Leuven, Belgium; bob.puers@esat.kuleuven.be

**Keywords:** EMG, sEMG, IMU, Bluetooth, BLE, biomedical, wearable

## Abstract

Due to a sedentary lifestyle, the amount of people suffering from musculoskeletal back diseases has increased over the last few decades. To monitor and cure these disabilities, sensors able to monitor the patient for long-term measurement during daily life and able to provide real-time feedback are required. There are only a few wearable systems that are capable to acquire muscle activity (sEMG) and posture at the same time. Moreover, previously reported systems do not target back sensor and typically comprise bulky uncomfortable solutions. In this paper, we present a new wearable sensor network that is designed to measure muscle activity and posture specialized for back measurement. Special care was taken to propose a discrete and comfortable solution. The prototype only measures 3.1 mm in thickness on the spine, making this sensor system the thinnest and lightest one in the literature to our best knowledge. After testing, it was shown that the sensor system is able to acquire two surface electromyography signals concurrently, to gather acceleration and rotation speed from the patient’s lower back, and to transmit data to a computer or a smartphone via serial communication or Bluetooth low energy for a few hours for later processing and analysis.

## 1. Introduction

With the improvement of life expectancy and large increase in sedentary lifestyle (office work, low physical activity) in the western countries over the last few decades, the number of patients suffering from back pain has strongly increased. According to Dieleman et al. [1], lower back and neck pain accounts for the third-largest condition of health care spending in the United States and represents approximately $87.6 billion of expense. Its lifetime prevalence in common cases is estimated between 60% and 70% in industrialized countries [2]. This situation has caused the WHO to highlight lower back pain as one of the priority diseases to focus on [2].

The medical procedure for lower back pain condition consists of two major phases:

- Diagnosis: Lower back pain can be induced by multiple risk factors. It is complex to measure the posture and movement adopted by the patient during his daily and working life (for example, posture and movement while lifting heavy loads, posture while sitting on a chair, repeated movement in a factory, etc.). It is indeed not trivial for a physician to measure the posture of a patient when the patient is not in a medical facility. Moreover, there could be a need to conduct the measurement over long periods (several days or weeks).
- Therapy: While pain can usually be symptomatically treated by medication, bad posture habits cannot. It usually takes time to turn bad habits into good habits. It is then critical for the therapist to have a mean to help the patient after the diagnosis which can require weeks and months of real-time monitoring and real-time notification to help the patient to learn new good habits about their postures and movements. As demonstrated by [3], home training of a patient is more effective when using sensor-based feedback than using a simple mirror or no feedback.

In both cases, the key point is to realize a device that is unnoticed and can be forgotten by the patient in order to let him/her behave in a natural way. Indeed, size, connectivity, or weight of the sensor system could lead to discomfort preventing the patient to reproduce his/her bad habit during the measurement or preventing to adapt to a new, good posture while in remission.

Multiple research groups (either from commercial companies [4,5,6,7,8,9] or research laboratories [10,11,12,13,14,15]) have been working on developing tools that can monitor body posture and/or record muscles activity in a wearable way. Most of the commercial companies follow the same strategy: they focus on offering a small independent sensor system powered by a battery that communicates wirelessly with a remote station (computer, smartphone, or dedicated electronics) and use an inertial motion unit (IMU) to estimate the posture. The focus typically is on designing the best sensor system either for specific applications or for general cases. One of the market leaders is Delsys [16], which provides a combined wearable system embedding a surface electromyography (sEMG) acquisition circuit and a nine-degrees-of-freedom (DoF) IMU, containing a three-axis accelerometer, a three-axis gyroscope, and a three-axis magnetometer. Both sensors are packaged in a rigid box as small as 27 mm × 37 mm × 13 mm. It weighs 14 g and can last up to 8 h. The company Cometa [17] focuses on providing separated dedicated sensors. This approach allows them to realize a wearable 9 DoF IMU package in a plastic box as small as 33 mm × 25 mm × 7 mm, weighing 10 g and lasting up to 8 h and a dedicated sEMG sensor measuring 40 mm × 15 mm × 10 mm with a weight of 7 g and a battery life of 12 h. Such sensor systems are useful for most athletes trying to optimize their movements or patient trying to recover from common injuries during exercise sessions with a therapist. However, they might not be suitable for other application scenarios. One obvious example is a typical office worker sitting 8 h per day and having to wear such a device permanently. Aforementioned systems are too bulky to be unnoticed, which is likely to lead to a modification of the natural posture or preventing the patient to learn good new habits. It is especially true when, for instance, the sensors are placed onto a vertebra of the spine.

Some research groups have focused on sensor systems dedicated for human back monitoring. In the same way as commercial companies, they use multiple sensors on the back and the head to estimate the position of the spine and muscle load over time. However, because of their volume, these sensor systems suffer from the same drawbacks as the aforementioned ones and are meant to be used by standing workers, working with heavy loads or repetitive movements. In 2019, Min-Su Song et al. [18] developed a thin flexible sEMG sensor that could potentially be used as a back sensor. The acquisition circuit measured 40 mm × 20 mm without packaging and battery, and was used for human–computer interaction (HCI) via Bluetooth. They did not mention the thickness of the final packaged prototype. In 2020, Jae Keun Lee et al. [19,20] developed a waterproof, stretchable, and wearable flexible six-DoF IMU circuit using thin copper foil package in a stretchable adhesive film (Tegaderm). Here also, they did not mention the total thickness of the sensor (100 µm for the PCB and packaging, without components and battery). In 2020, Shing-Hong-Liu [21] proposed a semi-flexible solution using three separated rigid PCBs connected by wires to acquire sEMG signals and detect muscle fatigue. However, the main unit contains only an accelerometer without gyroscope and only one sEMG channel. Therefore, it can be concluded that previously described sensor systems for back monitoring are not very suitable for applications in which long-term measurements in daily routine activities are required.

In this paper, we present a novel system that is able to acquire multiple biomedical data concurrently (posture via body movements and muscle activities) and send them to a computer/smartphone for real-time monitoring. Special attention is given to the size of the final product in order to interfere as little as possible with natural patient posture by reducing its thickness to a minimum so that it is suitable for long-term posture monitoring of patients that are, among others, sitting or lying.

## 2. Materials and Methods

The system consists of three units distributed over 3 subparts:

1. Unit 1 is intended to acquire the muscles activity via two surface electromyography (sEMG) measurement circuits. One on the left and one on the right side of the body.
2. Unit 2 comprises a 6-DoF IMU measuring linear accelerations and rotation speeds in three axes each to estimate the position and orientation of the subject of interest.
3. Unit 3 is the main electronic unit composed of processing, communication and power management.

**Figure 1:**
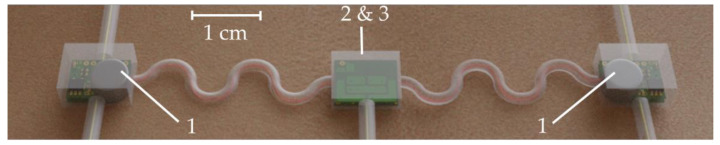
CAD illustration of the sensor system: the two outer blocks are the sEMG measurement circuits and the middle one contained the 6-DoF IMU and the main electronic unit.

Figure 1 shows a CAD illustration of the sensor system.

### 2.1. Surface Electromyography Sensor

The sEMG circuit contains the necessary amplification and filtering stages to obtain the highest amplitude signal (without saturation) before being transmitted to the centralized unit via an embedded 10-bit ADC. As the sEMG signal is affected by the patient anatomy [22,23,24], the muscle targeted and the electrodes impedance or the type of gel, it is difficult to determine general gain values for each sEMG sensor. However, it is known that sEMG signals typically range from 0 to 10 mV [25] and, therefore, a total gain of approximately 1500 *v*/*v* (see below) was chosen.

**Figure 2:**
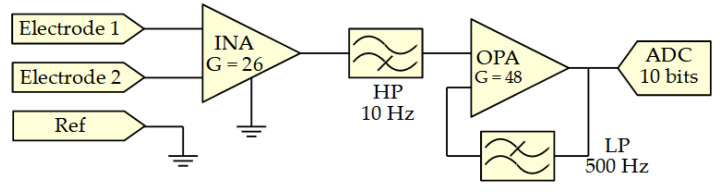
Simplified schematics of the sEMG acquisition circuit.

The sensor interface consists of a differential amplification stage with a first gain of 26 *v*/*v* followed by a high pass filter with 10-Hz cut-off frequency [26]. This removes any DC component in the signal which could induce saturation in the second amplification stage. This DC component in the signal is the reason why the first gain has to be limited whereas it is usually recommended to have the first gain as high as possible to minimize added thermal noise. An INA333 [27] instrumentation amplifier (INA) from Texas Instruments was selected as a good tradeoff between low quiescent current (50 µA), low noise (50 nV/√Hz), and a “very-very-thin small-outline no-lead” (WSON) package measuring 3 mm × 3 mm × 0.75 mm. It is followed by an active low pass filter with a cut-off frequency of 500 Hz [26] and a secondary gain of 48. An OPA379 [28] operational amplifier (OPA) from Texas Instruments was selected as a good tradeoff between low quiescent current (5.5 µA), matching gain bandwidth product (90 kHz) and small SC70-5 [28] package measuring only 2 mm × 2.1 mm × 0.95 mm. A simplified schematic of the sEMG acquisition circuit is shown in Figure 2.

### 2.2. Inertial Sensor

An ICM-42605 [29] from the company TDK—InvenSense was chosen as the inertial measurement unit. It embeds an accelerometer measuring the acceleration along the three axes as well as a gyroscope measuring the rotation speed of the sensor around the three axes. The chip was chosen for its tiny footprint (2.5 mm × 3 mm × 0.91 mm) and low consumption of only 0.65 mA in worst case (low-noise mode). It also has the advantage to minimize the number of required surrounding components to only three resistors and capacitors. The setup properties of the sensors are detailed in Table 1.

A 9-DoF IMU could have been used in this project but would have significantly increased the required current drain from 0.65 mA to 3.11 mA [29,30]. Moreover, other studies [31,32] have demonstrated that a magnetometer is not required to estimate human body postures. While most human movements in postural studies range from 1 to 5 Hz [33], state-of-the-art sensors use an IMU update rate ranging from 25 Hz to 250 Hz [9,12,17,19,33,34]. As shown in Table 1, 125 Hz and 100 Hz were selected in this work, based on the limited available output data rates available from the chip’s datasheet.

### 2.3. Main Unit

The main unit is the scheduler of the sensor system. It is in charge of collecting and digitalizing the sEMG, requesting the motion data from the IMU, and sending them to external devices.

**Figure 3:**
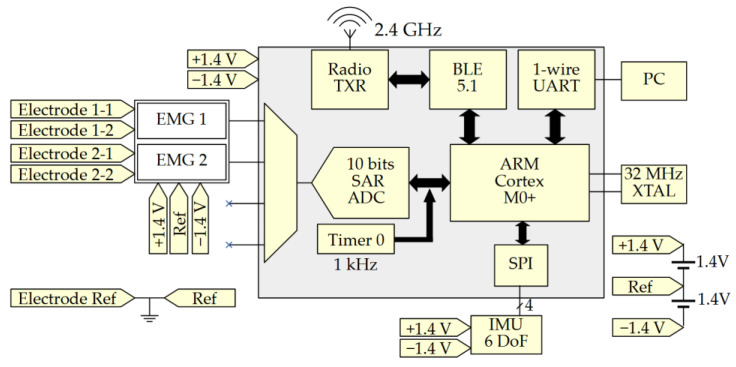
Block diagram of the main processing unit.

This part of the system comprises a low power DA14531 [35] chip from dialog semiconductor measuring 2.2 mm × 3 mm × 0.4 mm. It is a Bluetooth low-energy (BLE) 5.1 system-on-chip, embedding an ARM Cortex M0+ central processing unit (CPU). It contains a 4-channel 10-bit successive approximation (SAR) analog to digital converter (ADC) to acquire the two sEMG streams at 1000 Hz. The DA14531 also embedded a serial peripheral interface (SPI) bus used to communicate with the IMU at 24 MHz with an acquisition rate of 100 Hz. The data collected by the MCU are sent via Bluetooth or universal asynchronous receiver-transmitter (UART) every 100 ms. The selection of communication protocol is carried out during programming of the chip and either one or both can be activated simultaneously. Finally, two hearing aid A10 batteries connected in series are used to power the overall system. These batteries using a zinc-air reaction have a capacity of 91 mAh and measure 5.8 mm in diameter with a thickness of 3.6 mm. Their nominal voltage varies between 1.4 V at maximum charge and 1.2 V on discharge. Figure 3 shows a block diagram of the main processing unit and the connection with surrounding electronics.

To reduce the load of the microcontroller, values collected from the sEMG sensors and the IMU chip are concatenated over time before being transmitted. The two sEMG outputs are sampled 1000 times per second as recommended by the SENIAM project (Surface ElectroMyoGraphy for the Non-Invasive Assessment of Muscles) [26,36] and added to two 16-bit arrays. The data from the IMU are represented by a table of 3 × 2 cells (3-axis acceleration and 3-axis gyroscope) of 16 bits and are sampled 100 times per second. This represents an amount of 41.6 kbits/s. Special care was taken to avoid latency in the acquisition process. A timer is used to schedule the process. In the case of an unsuspected event resulting in the ADC acquisition or the SPI communication with the IMU being blocked, the algorithm was written so that it interrupts the problematic acquisition instance and duplicates the previous value.

### 2.4. Wireless Communication

**Figure 4:**
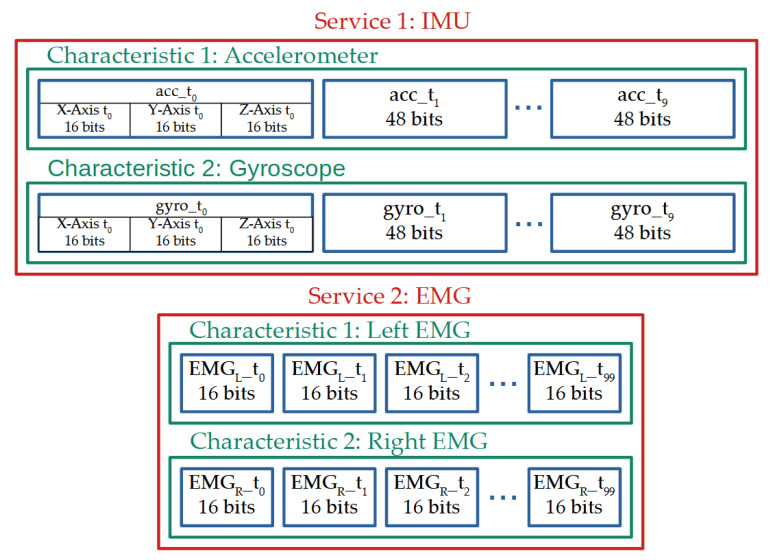
Bluetooth packet decomposition split into two services each split in two characteristics updated at 10 Hz.

The transmission of the data is performed using the Bluetooth low-energy protocol implemented by Dialog Semiconductor on the DA14531 chip. Two services were created: one for the IMU and one for sEMG acquisition circuits. Figure 4 shows a summary of the Bluetooth communication organization.

The first service, used to transmit the ten concatenated IMU values, contains two characteristics updated at a rate of 10 Hz. Each characteristic is dedicated to a sub-sensor of the IMU (the accelerometer or the gyroscope) and contains the 16-bit values of the three axes concatenated together over time, resulting in a stream of 960 bits length. The second service, used to transmit the hundred concatenated sEMG values, contains two characteristics also updated at a rate of 10 Hz. Each characteristic is dedicated to one of the two sEMG sensors and contains the sEMG 16-bit values concatenated over time, resulting in a stream of a 3.2 kbits length. Both services sum up to a transmission packet of 4.16 kbits.

In theory, BLE 5.0 and more recent versions can transmit data up to 2 Mbits/s [37]. The chip selected for the project is limited to 1 Mbits/s. This project data rate is way below this limit, allowing it to resend packets in case of transmission failures, in theory. In this case, the 10 Hz updating rate was chosen in order to optimize the power consumption while reducing the latency for real-time display (see Section 3.5).

### 2.5. Sensor System Fabrication

**Figure 5:**
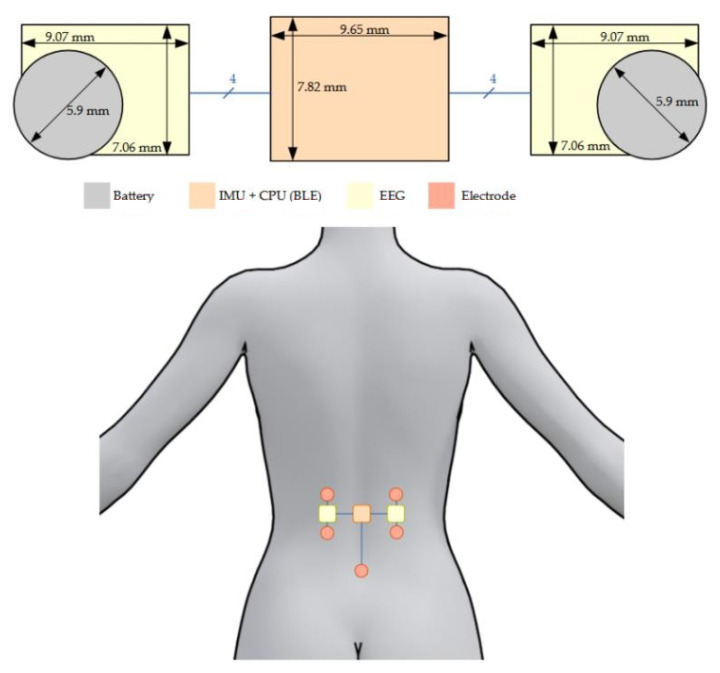
Position, shape and distribution of the hardware over the three areas.

Standard FR4 PCBs are rigid and have a thickness of 1.55 mm. Although the FR4 PCBs could have been produced with a thickness of 0.1 mm minimum, the final sensor would have been rigid, increasing the discomfort of the patient. Therefore, a flexible polyimide material of only 50 µm was used for the PCBs. The total thickness of the PCB including polyimide substrate (50 µm) and two copper layers (2 × 18 µm) was 86 µm. The sensor was divided into three separated areas: (i) a central area located on the spine containing the IMU and the CPU (with Bluetooth); and (ii) two decentralized areas containing each an sEMG acquisition circuit and a battery, as shown in Figure 5. Each area was connected via 4 wires providing power (+1.4 V, Ref and −1.4 V) and the amplified analog signal.

**Figure 6:**
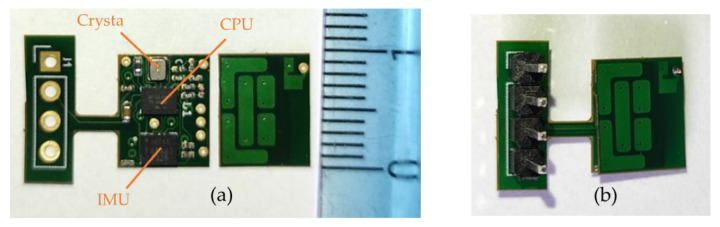
PCBs of the central area. (**a**) The two separated PCBs that will be stacked: on the left the CPU, the IMU, and the crystal; on the right the ZOR antenna. (**b**) The result when the two layers are stacked.

**Figure 7:**
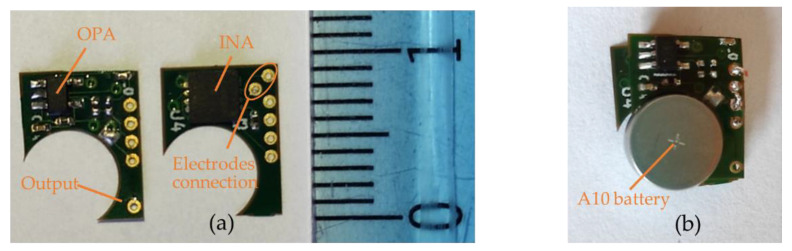
PCBs of the decentralized area. (**a**) The two separated PCBs: on the left the operational amplifier with filters and the output pad; on the right the instrumentation amplifier with electrode connection points. (**b**) The result when the two layers are stacked and when one battery is placed.

1. The central area, placed on the spine, was designed to be as thin as possible while maintaining a reasonable surface. It contains the CPU, the IMU, a crystal, and few passive components with the following respective thicknesses: 400 µm, 910 µm, 650 µm, and 350 µm. This brings a final theoretical PCB thickness of 996 µm with a surface of 9.65 mm × 7.82 mm (without antenna). Despite the fact that we used a zeroth-order resonator (ZOR) antenna measuring only 8.97 mm × 6.27 mm, connecting the antenna next to the PCB would have almost doubled its size. As a tradeoff between size, thickness, and emission performance, it was decided to stack the antenna onto the PCB providing a final sensor measuring 9.65 mm × 7.82 mm × 1.09 mm. Figure 6 shows photos of the PCB, the antenna, and the result when the two PCBs are stacked. The left part of the PCB containing the 4-pin headers is meant to be cut once the CPU is programmed and the prototype is ready to be used.
2. The two decentralized parts, placed a few centimeters from the center of the back and on the side, were designed to be as compact as possible. They were designed symmetric from each other. Each part contained an INA, an OPA, and few passive components having the following respective thicknesses: 750 µm, 950 µm, and 250 µm. Since the battery was the biggest element measuring 3.6 mm in thickness, it was decided to split the sEMG acquisition circuit into two PCBs and stack them to reduce its footprint. This resulted in two stacked circuits with a footprint of 25 mm^2^ instead of 43 mm^2^ and a thickness of 1.872 mm instead of 0.996 mm. The final area including the battery measured 9.45 mm × 7.27 mm × 3.6 mm. Figure 7 shows pictures of the two layers and the combined parts with the battery.

### 2.6. Packaging and Connection

**Figure 8:**
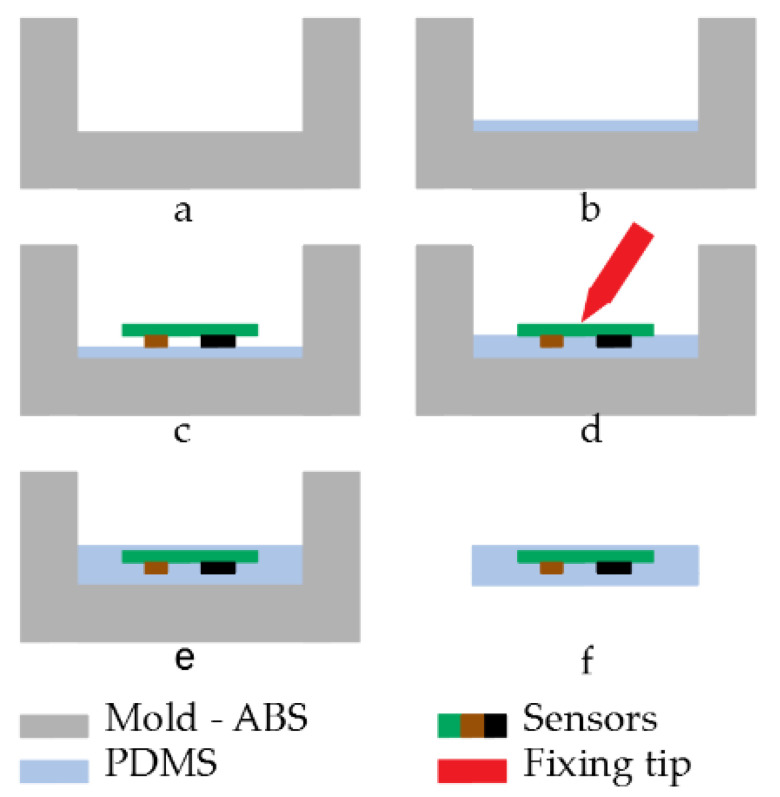
Packaging process: (**a**) design of the ABS mold, (**b**) addition of a first PDMS layer, (**c**) placement of the PCB, (**d**) locking of the PCB position, (**e**) filling of the mold, (**f**) unmolding of the PCB.

Both areas of the sensor system had to be protected again contacts, sweat, shocks, dust, etc., which could damage the sensor or lead to malfunctioning. To keep parts of the PCB flexible and to increase the comfort of the patient, rigid packaging was not an option. For these reasons, a polydimethylsiloxane (PDMS) SYLGARD™ 184 package was developed. Each part packaging followed the same principle:

1. An acrylonitrile butadiene styrene (ABS) mold with the desired shape was created using 3D printing technology (Figure 8a). ABS was selected over polylactic acid (PLA) for its higher glass transition temperature which is around 105 °C despite its higher complexity to 3D print;
2. A first layer of PDMS was deposited onto the bottom of the mold. This prevented a contact between sensors and mold and thus an incomplete sealing (Figure 8b);
3. Once the first layer was cured, the sensor system was placed and maintained in position while a second layer of PDMS was added. This layer was used to fix the sensors in its final position and to prevent them from moving during the final step (Figure 8c,d). Special care was taken to prevent the sensors from touching the mold and create a failure in the sealing;
4. Once the second layer was cured and the PCB fixed in the correct position, the mold was fully filled with PDMS (Figure 8e);
5. The packaged sensors were removed from the mold (Figure 8f).

It was critical to use a degassing process for each step including a new PDMS layer. Not doing this would result in bubbles stuck in the PDMS resulting in a mechanical weak point or an opening in the sealing.

## 3. Results

A complete set of tests was conducted to validate the sensor. First, the sEMG function and the IMU were validated on their own. Then, a long-term measurement was conducted to validate the use of the sensor system for long period. Finally, the packaging and the communication were analyzed.

**Figure 9:**
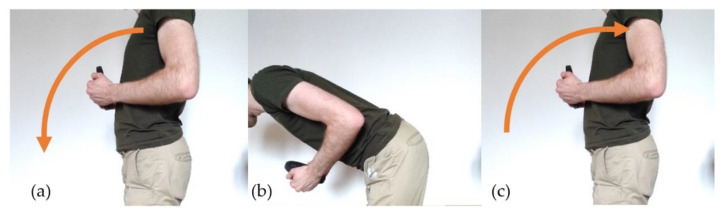
Procedure followed for sEMG and IMU test setup. (**a**) Bend forward. (**b**) Stay in position. (**c**) Move back to the initial position.

During the test, each part of the system was tested separately with the same test procedure to verify its correct behavior. During the test, the subject was asked to follow the following procedure:

1. Bend forward while carrying a 3 kg dumbbell (Figure 9a);
2. Stay in position for approximately 1 s (Figure 9b);
3. Move back to initial (straight) position (Figure 9c).

### 3.1. Surface Electromyography Sensor

**Figure 10:**
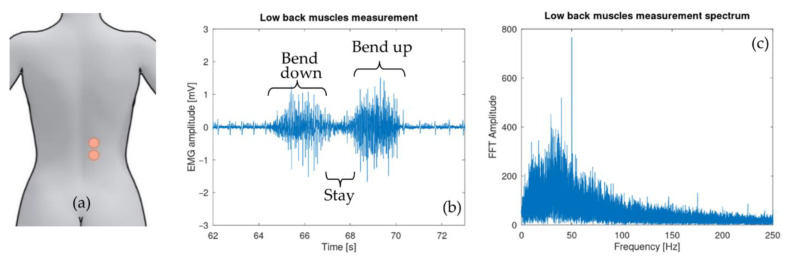
(**a**) Differential electrode placement. (**b**) Lower back muscle activity during exercise: bending forward, staying in position, moving back to the initial position. (**c**) Fast Fourier transform of the collected sEMG signal. This shows the usual sEMG spectrum with a reasonable 50 Hz contamination.

Electrodes were attached to low back muscle on a 29-year-old test subject with low subcutaneous fat amount (BMI around 20). They were placed on the erector spinae muscle at the height of the second lumbar vertebra L2 a few centimeters on the right side (Figure 10a). Figure 10b shows the sEMG signal after being digitalized and sent by the microcontroller. The spectrum of the acquired signal was also computed via the FFT algorithm provided by the GNU octave software [38].

### 3.2. Inertial Sensors

**Figure 11:**
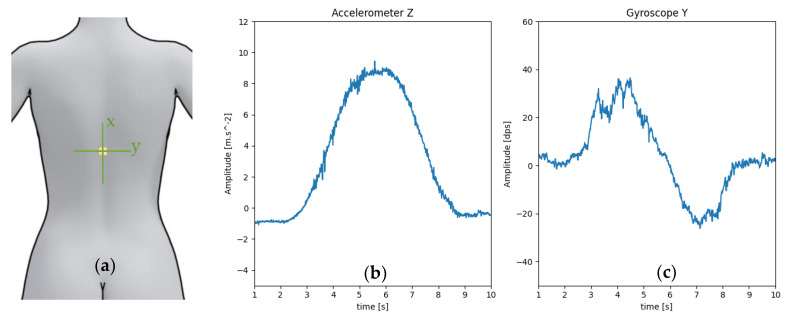
(**a**) Position of the IMU during the test with its axes. (**b**) Acceleration along the *z*-axis, maximum when the test subject back is parallel to the floor. (**c**) Rotation speed around the y axis of the test subject back: positive when bending down, negative when coming back to the initial position.

The IMU was attached to the skin in the middle of the back at the height of the eighth thoracic vertebra T8 (Figure 11a). Over the six IMU signals acquired, the two relevant ones with regard to the test (*z*-axis accelerometer and *y*-axis gyroscope) are shown in Figure 11b,c.

### 3.3. Long-Term Measurement

**Figure 12:**
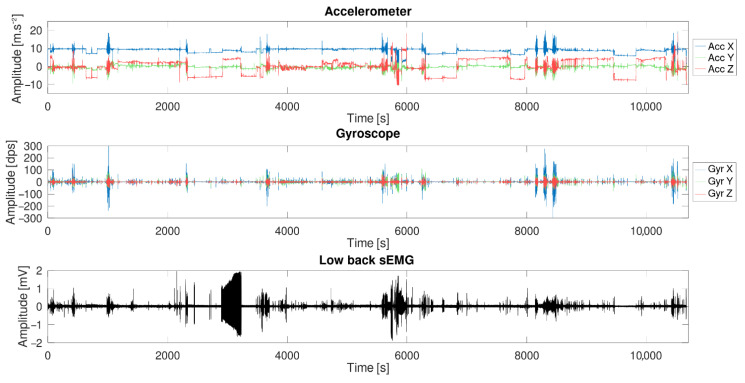
Long-term measurement (3 h) made with the sensor during a normal day: the complete acquisition data from one sEMG sensor and the inertial sensor.

The sensor was placed on the test subject for a period of 3 h and both inertial sensors and surface electromyography data were recorded. The subject was asked to behave as he was used to do normally during evenings (sits on a chair/sofa, works on a computer, workout, etc.). The electrodes were placed on the erector spinae muscle at the height of the lumbar vertebra L3 and L1, and the six-DOF IMU was placed at the height of the L2 vertebra. Figure 12 shows the result of the test.

### 3.4. Packaging and Connection

**Figure 13:**
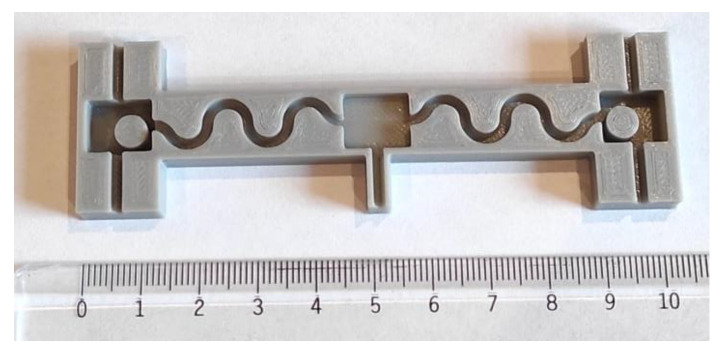
The ABS 3D printed mold used to package the sensor and the wires connecting each subpart. The two pillars in the external areas are meant to keep battery spaces clean of PDMS. This allows easy placement and replacement of the batteries.

Since movements of the patient might lead to stretching of the back skin, the sensor system should also be able to be stretched. While PDMS can be stretched, electrical wires cannot. For this reason, wires were packaged in a wavy shape. Moreover, it was important to have wires as flexible as possible. A cable composed of stranded wires with a diameter of 0.06 mm and coated with Teflon (polytetrafluoroethylene, PTFE) was adopted. The 3D printed mold is shown in Figure 13.

**Figure 14:**
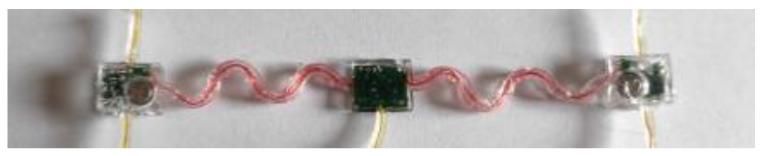
Final sensor: the three subparts (left sEMG, main unit and right sEMG) packaged in PDMS with the two batteries included. Vertical wires are connected to electrodes.

Once each layer was added, degassed, and properly cured, the sensor was unmolded carefully to avoid any damage to the packaging. Figure 14 shows the final packaged sensor system.

**Figure 15:**
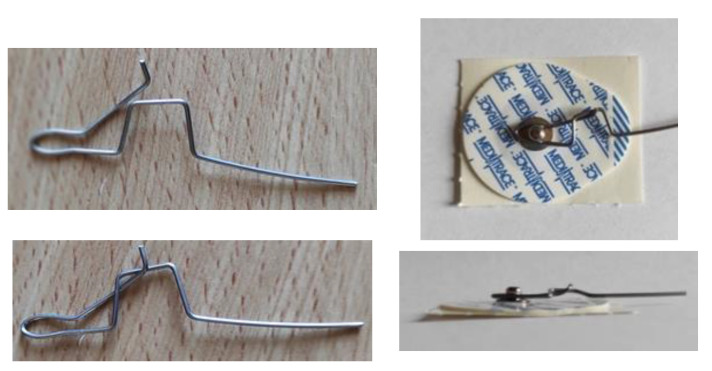
Thin clamping system developed to connect the electrode to the sensor without affecting the final thickness.

The standard way to connect disposable electrodes to patients is achieved via snap lead wire adapters, alligator clamps, or pre-wired electrodes. The drawback of these solutions is their thickness. The snap adapters are 6 mm thick and, once connected to the electrodes, the final assembly (electrode and connector) is 8 mm thick. The alligator clamps vary with different models, but because of the way they operate, it is not suitable for this project. There is a risk of opening the clamp if the patient sits against the backrest of a chair or lies on a bed. Finally, the pre-wired electrodes might seem to be a good solution, but they require a DIN-female connector measuring around 1 cm in diameter. For these reasons, a homemade system was developed and is shown in Figure 15. It consists of a 1-mm-diameter steel wire folded in the shape of a clamp with a locking system. The clamp is made to curl around the electrode tip. By using this connector, the final assembly is 4 mm thick, which is the thickness of the electrode.

**Figure 16:**
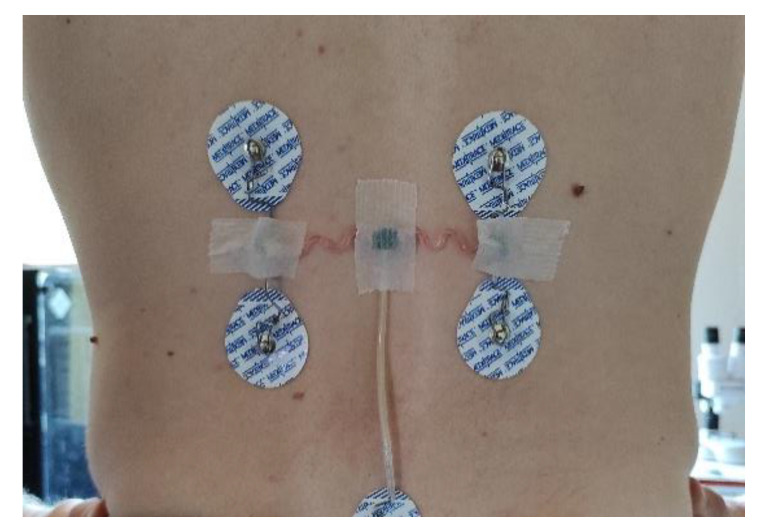
Final packaged sensors stuck to a test subject using medical tape.

To attach the sensor to the patient, a medical tape is placed over the three rectangle subparts to stick the sensor on the back of the patient. Wavy arms containing wires remain free to move and to stretch. Figure 16 shows the sensor placed on the back of a test subject.

### 3.5. Communication

**Figure 17:**
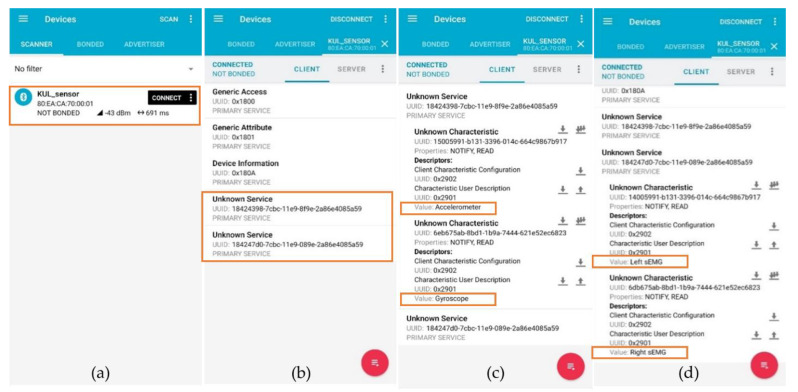
Screenshot of the “nRF connect” application [39] running on an android smartphone connection to the sensor system. (**a**) Detection of the sensor system. (**b**) The two services (IMU and sEMG) are detected. (**c**) The first service (IMU) contains the two characteristics representing the accelerometer and gyroscope value. (**d**) The second service (sEMG) contains the two characteristics representing the left and right sEMG values.

Two different communication techniques were validated independently during the test: a wired solution using the universal asynchronous receiver transmitter (UART) protocol at a 115,200 baud rate, transmitted to a computer via a USB cable; and a wireless solution using the Bluetooth low-energy (Bluetooth 5.1, 2.4 GHz) protocol, transmitted to a smartphone using the “nRF connect” application from Nordic Semiconductor [39]. Figure 17 shows the two services and the four characteristics viewed by the smartphone.

To select the ideal packet load and transmission frequency, a test comparing transmission error/maximum rate in function of the two previous variables was conducted for BLE. The results are shown in Table 2.

## 4. Discussion

### 4.1. Surface Electromyography Sensor

**Figure 18:**
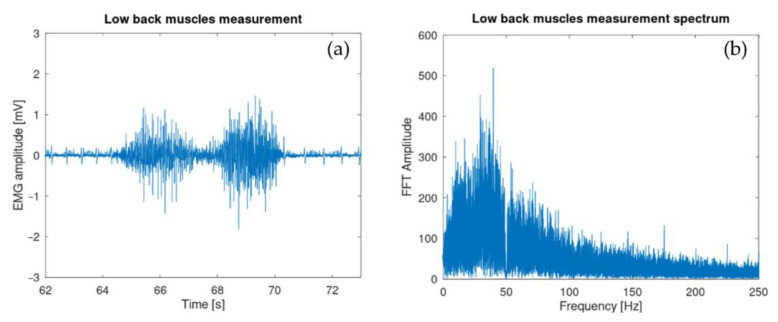
Effect of a post-process band stop filter (2nd order Butterworth) with a cut-off frequency from 49 Hz to 51 Hz. (**a**) Time domain and (**b**) frequency domain signals.

As seen in Figure 10b, the three phases of the test are clearly visible. The signal was corrupted by an electrocardiogram (ECG) signal partially filtered by the 10-Hz high-pass filter. This contamination is a well-known effect that arises particularly during measurements of trunk muscles. Multiple techniques exist in order to remove this ECG signal [40]. It is also important to have a look at the fast Fourier transform (FFT) (see Figure 10c) of the sEMG signal to see that the shape is similar to the usual sEMG spectrum. It should be noticed that the signal is reasonably contaminated by 50-Hz noise. This parasitic signal could be removed using post-processing filters although it is not recommended since it would affect the sEMG signal itself [26]. Figure 18 shows the effect on the time domain and spectrum that a post-process band stop filter (2nd order Butterworth) with a cut-off frequency from 49 Hz to 51 Hz would produce.

### 4.2. Inertial Sensors

As seen in Figure 11b, the acceleration along *z*-axis did not start at 0 as it should do for a perfectly vertical position. This was because the human back is naturally curved. Moreover, the test subject was standing in a natural position which places the sensor in a non-perfect vertical position. The initial and final (t = 10 s) acceleration signals were slightly different from each other for a similar reason: the test subject did not finish at exactly the same position as the initial one.

The curve shown in Figure 11c was not smooth as it would be expected for simple bending movement. This can be explained by the slow movement of the test subject while carrying a charge inducing small vibration during the movement and, thus, irregular rotation speed shape.

While acceleration rate and rotation speed are not directly useable by a therapist, it was shown that they can be post-processed to estimate the posture of the owner. Previous work [41,42] has already demonstrated that these data can be used to discriminate some specific movement performed by the owner. This movement classification is left to partner teams that will develop a mobile health application based on previous studies (see conclusion).

### 4.3. Long-Term Measurement

**Figure 19:**
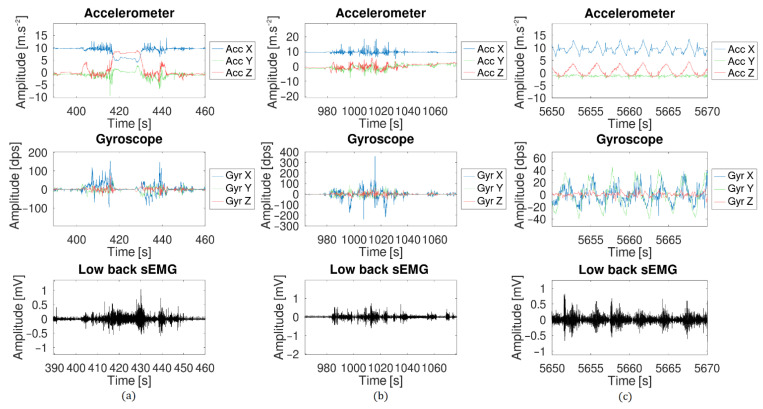
Zoom of three events from the long-term measurement: (**a**) the test subject bent forward to look in a drawer, (**b**) the test subject walking in his house, (**c**) the test subject did a few squats.

The long-term measurement provided good results although problems arose. Figure 19 shows three zoomed events selected from the complete measurement data. During the first one (Figure 19a), the test subject bent forward to find an object in a drawer. During the second event (Figure 19b), the test subject was walking in the house. Finally, the last event came from a stretching session during which one the test subject did a few squats.

**Figure 20:**
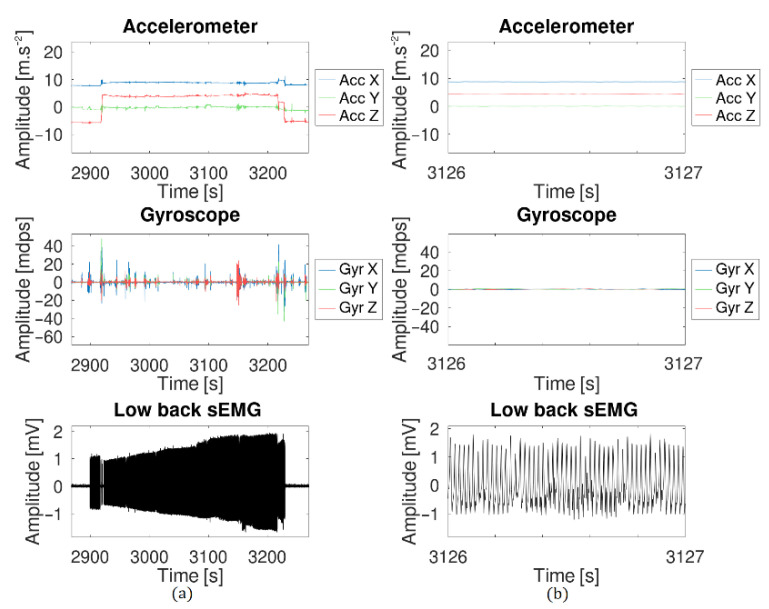
Effect of the reference electrode being disconnected. The 50-Hz noise completely hides the sEMG signal. (**a**) Global view of the effect. (**b**) Zoomed version to have a better view of the 50 Hz signal.

As already seen in the previous experiment, the sEMG signal was corrupted by the ECG signal and a relatively low 50-Hz noise. During the session, the test subject accidentally pulled off the reference electrode. The result is shown in Figure 20. When the reference electrode was disconnected, a strong 50 Hz noise corrupted the signal and the initial sEMG was not visible anymore. Special care must be taken when electrodes are selected and placed to avoid this situation.

More complete testing of the sensor system with patients suffering from a variety of rheumatic and musculoskeletal diseases would be interesting in order to determine which specific diseases can be targeted with the sensor system proposed in the paper.

### 4.4. Packaging and Connection

The sensor developed is the thinnest one available in the literature (measuring 3.1 mm thick on the spine and 5.6 mm thick on the side of the spine). The final size of the total prototype (two sEMG sensors, a central unit with IMU and the intra sensors wires connections) is 100 mm × 10 mm × 6 mm and the total weight is 2.9 g. Table 3 and Table 4, respectively, show a comparative summary of this work with state-of-the-art works (either commercial or research).

The estimated price of the complete packaged sensor is estimated around EUR 10 (without battery and electrode) for a production volume of 1000 units. Commercial products usually cost a few hundred euros to buy, which makes the sensor system developed in this work a cheaper solution. However, commercial product prices also include R&D cost and profit margin, which were not taken into account in the computation for the price of this sensor system.

### 4.5. Communication

As it was shown in Table 2, the maximum wireless throughput was limited to 16.8 kbits/s (2.1 kBytes/s). Further development of the code will be required to improve this limit and to ensure a complete and correct transmission of all sensor data simultaneously. In order to reduce the power consumption, preprocessing of the data could be carried out with the MCU to reduce the amount of data that need to be transmitted. Most of the sensors cited in Table 3 proposed sensor fusion data as an output at a lower sampling rate. Depending on the application targeted, characterization of the movement could be carried out on the MCU. This would drastically reduce the needed amount of data to be transmitted and, thus, the power. Currently, the system sends the data using the standard Bluetooth low-energy protocol. To avoid any privacy concerns, it is important to use an encrypted communication proposed by BLE [43] when used on a real patient in daily life.

## 5. Conclusions

In this paper, a new sensor system was proposed to address specific needs of therapists. While some companies and research groups have already developed sensors able to measure limbs position and muscles activity, none have proposed a sensor system dedicated to (lower) back measurement for daily activities (office work, sitting on a chair, lying on a bed, etc.).

The comparison presented in Table 3 and Table 4 show that miniaturization was successfully achieved but sampling rate and resolution were reduced as a tradeoff. However, despite this reduction, the sensor characteristics remain in the required specifications recommended by professionals or other research groups.

In further work, embedded data processing, such as fusion algorithm and movement discrimination for the IMU, correlated with the sEMG data, should be implemented in order to reduce the data transmission load and reduce the power consumption. Moreover, having this pre-processed data would allow a dedicated smartphone application to warn the patient of harmful movements or positions. Finally, a mobile health application will be developed to follow the patient’s status. This application will record logs for the therapist (to diagnose and update the disease status) and will be programmable to recognize specific repetitive movements and postures that should be avoided by the patient (and trigger some notifications based on the therapist’s input). Studies have been conducted to validate the need for such mobile applications [44].

## Figures and Tables

**Table 1:** ICM-42605 setup properties.

Properties	Accelerometer	Gyroscope
Resolution (x, y, z)	14 bits	16 bits
Bandwidth	125 Hz	100 Hz
Range	±16 g	2000 dps
Noise	70 µg/√Hz	3.8 mdps/√Hz

**Table 2:** Comparison of the effect of update frequency and packet size on the Bluetooth low-energy maximum transmission rate. The three best rates are highlighted in yellow. Value 0 represents a too high payload leading to disconnection of the sensor.

	Packet Size (Bytes)																							
Update Frequency [Hz]		1	2	3	4	5	6	7	8	9	10	20	30	40	50	60	70	80	90	100	200	300	400	500
	1	1	2	3	4	5	6	7	8	9	10	20	30	40	50	60	70	80	90	100	200	300	400	0
	2	2	4	6	8	10	12	14	16	18	20	40	60	80	100	120	140	160	180	200	400	600	800	0
	3	3	6	9	12	15	18	21	24	27	30	60	90	120	150	180	210	240	270	300	600	900	1200	0
	4	4	8	12	16	20	24	28	32	36	40	80	120	160	200	240	280	320	360	400	800	1200	0	0
	5	5	10	15	20	25	30	35	40	45	50	100	150	200	250	300	350	400	450	500	1000	1500	0	0
	6	6	12	18	24	30	36	42	48	54	60	120	180	240	300	360	420	480	540	600	1200	1800	0	0
	7	7	14	21	28	35	42	49	56	63	70	140	210	280	350	420	490	560	630	700	1400	2100	0	0
	8	8	16	24	32	40	48	56	64	72	80	160	240	320	400	480	560	640	720	800	1600	0	0	0
	9	9	18	27	36	45	54	63	72	81	90	180	270	360	450	540	630	720	810	900	1800	0	0	0
	10	10	20	30	40	50	60	70	80	90	100	200	300	400	500	600	700	800	900	1000	2000	0	0	0
	20	20	40	60	80	100	120	140	160	180	200	400	600	800	1000	1200	1400	1600	1800	2000	0	0	0	0
	30	30	60	90	120	150	180	210	240	270	300	600	900	1200	0	0	0	0	0	0	0	0	0	0
	40	40	80	120	160	200	240	280	320	360	400	800	1200	0	0	0	0	0	0	0	0	0	0	0
	50	50	100	150	200	250	300	350	400	450	500	1000	0	0	0	0	0	0	0	0	0	0	0	0
	60	60	120	180	240	300	360	420	480	540	600	1200	0	0	0	0	0	0	0	0	0	0	0	0
	70	70	140	210	280	350	420	490	560	630	700	1400	0	0	0	0	0	0	0	0	0	0	0	0
	80	80	160	0	0	0	0	0	0	0	0	0	0	0	0	0	0	0	0	0	0	0	0	0
	90	0	0	0	0	0	0	0	0	0	0	0	0	0	0	0	0	0	0	0	0	0	0	0

**Table 3:** Summary and comparison of this work to other sensors, focusing on the IMU. The best characteristics are highlighted in green.

	This Work (IMU)	Shimmer [4]	FreeMG [9]	Brunelli et al. [15]	Trigno [16]
Length [mm]	11.65	65	41.5	27	27
Height [mm]	9.27	32	24.8	18	37
Thickness [mm]	5.6	12	14	9.2	13
Number of channels	1	2	1	32	1
Resolution [bit]	10	NA	16	12	16
Max sample rate [Hz]	1000	8400	1000	1000	4370
Weight [g]	0.5	31	13	NA	7
Packaging	Soft PDMS	Rigid plastic	Rigid plastic	NA	Rigid plastic

**Table 4:** Summary and comparison of this work to other sensors, focusing on EMG. The best characteristics are highlighted in green.

	This Work (EMG)	LPMS-B2 [7]	Valero et al. [11]	Trigno [16]	Lee et al. [19]
Length [mm]	11.45	39	60	27	40
Height [mm]	9.82	39	40	37	37
Thickness [mm]	3.1	8	15	13	NA
DOF	6	9	NA	6	6
Resolution [bit]	10	NA	NA	16	16
Max sample rate [Hz]	100	400	50	963/741	100
Weight [g]	0.9 (each)	12	NA	10	NA
Packaging	Soft PDMS	Rigid plastic	Rigid plastic	Rigid plastic	Adhesive film

## Data Availability

Data available on request due to privacy restrictions.
